# A process evaluation of performance-based incentives for village health workers in Kisoro district, Uganda

**DOI:** 10.1186/1478-4491-12-19

**Published:** 2014-04-08

**Authors:** James S Miller, Sam Musominali, Michael Baganizi, Gerald A Paccione

**Affiliations:** 1 Harvard Medical School, 260 Longwood Avenue, Boston, MA 02215, USA; 2 Doctors for Global Health, PO Box 247, Kisoro, Uganda; 3 Kisoro District Hospital, Kisoro, Uganda; 4 Albert Einstein College of Medicine, 1300 Morris Park Avenue, New York, NY 10461, USA; 5 Montefiore Medical Center, 111 East 210th Street, New York, NY 10467, USA

**Keywords:** Developing countries, Incentives, Low-income countries, Primary health care, Uganda

## Abstract

**Background:**

Designing effective incentive systems for village health workers (VHWs) represents a longstanding policy issue with substantial impact on the success and sustainability of VHW programs. Using performance-based incentives (PBI) for VHWs is an approach that has been proposed and implemented in some programs, but has not received adequate review and evaluation in the peer-reviewed literature. We conducted a process evaluation examining the use of PBI for VHWs in Kisoro, Uganda. In this system, VHWs are paid based on 20 indicators, divided among routine follow-up visits, health education activities, new patient identifications, sanitation coverage, and uptake of priority health services.

**Methods:**

Surveys of VHWs (n = 30) and program supervisors (n = 7) were conducted to assess acceptability and feasibility. Interviews were conducted with all 8 program supervisors and with 6 purposively selected VHWs to gain a deeper understanding of their views on the PBI system. Program budget records were used to assess the costs of the program. Detailed payment records were used to assess the fairness of the PBI system with respect to VHWs’ gender, education level, and village location.

**Results:**

In surveys and interviews, supervisors expressed high satisfaction with the PBI system, though some supervisors expressed concerns about possible negative effects from the variation in payments between VHWs and the uncertainty of reward for effort. VHWs perceived the system as generally fair, and preferred it to the previous payment system, but expressed a desire to be paid more. The annual program cost was $516 per VHW, with each VHW covering an average of 115 households. VHWs covering more households tended to earn more. There was some evidence that female gender was associated with higher earnings. Education level and proximity to the district hospital did not appear to be associated with earnings under the PBI system.

**Conclusions:**

In a one-year pilot of PBI within a small VHW program, both VHWs and supervisors found the PBI system acceptable and motivating. VHWs with relatively limited formal education were able to master the PBI system. Further research is needed to determine the long-term effects and scalability of PBI, as well as the effects across varied contexts.

## Background

Village health workers (VHWs) have often been proposed as a central component of primary health care programs in developing countries [1-4]. The decision of how to incentivize VHWs represents a longstanding policy issue within the field of primary health care and has substantial impact on the success and sustainability of VHW programs. Providing proper incentives (whether monetary or non-monetary) not only provides motivation to work but also enables programs to retain VHWs and avoid the disruption caused by widespread attrition [5]. Some programs eschew monetary incentives out of a concern that the extrinsic motivation provided by money threatens to eliminate intrinsic motivations, such as a desire to help or a sense of religious duty [6]. Others, however, suggest that intrinsic motivations alone may not be enough to sustain high-functioning VHW programs, especially in places where high levels of disease burden and poverty increase the opportunity cost of volunteering one’s time – the exact settings in which VHWs are most needed [7]. However, monetary incentives can pose a challenge to program administration. Ensuring that payments are distributed to VHWs in an accountable and fair manner can be difficult. Using performance-based incentives (PBI) for VHWs is an approach that has been proposed and implemented in some programs, but has not received adequate review and evaluation in the peer-reviewed literature. We conducted a process evaluation of the implementation of PBI in a small VHW program in Kisoro, Uganda. A process evaluation seeks to examine issues such as acceptability, adherence, feasibility, quality of implementation, coverage, mechanism of action, and contextual influences or generalizability. These issues play a large role in the success of public health programs, particularly those involving multi-dimensional health promotion activities, but are often neglected in outcome evaluations [8].

Previous research on incentives for VHWs offers diverging conclusions about the role of non-monetary and monetary incentives. Prior to initiating this process evaluation, we conducted a literature search and identified and reviewed 9 studies assessing VHW motivations conducted in the past 10 years in a range of locations (Ethiopia, Malawi, South Africa, Kenya, Nigeria, Bangladesh, Nepal, and Mexico). Of these, 7 evaluated fully volunteer programs in which VHWs did not receive any monetary incentives, while 2 evaluated programs in which VHWs retained a volunteer identity but received some monetary incentives. Of the 7 evaluations of fully volunteer programs, 4 identified intrinsic motivations as both VHWs’ main reason for participation and as effective in maintaining continued participation [6,9-11]. Only one of these evaluations explicitly examined attrition, finding it to be under 5% per year [6]. Two of these studies suggested that spirituality or religion was a major source of intrinsic motivation [6,10]. It is also worth noting that one of these evaluations [6], while methodologically sound, primarily examined the perceptions of decision makers rather than VHWs, and has thus been criticized as potentially biased [12]. Three evaluations of fully volunteer programs reported high dissatisfaction with unpaid volunteer work, which led to high levels of attrition. One evaluation reported attrition of 22% over a 1-year period [13], while another reported 33% attrition over only 11 months [14]; the third reported attrition in an ambiguous manner without specifying a time period [15]. In one program, the main motivation for joining was the hope of eventual paid employment [13]; in all three, VHWs who had left the program reported that the lack of monetary incentives was the main reason for their departure [13-15]. However, the two evaluations of VHWs in paid programs also reported significant dissatisfaction among VHWs, as the payment amounts were perceived to be too low [16,17]. In both programs, hope for better-paid work in the future was a key motivation for sustained participation [16,17]. We hypothesized that a PBI system might fulfill the twin goals of maintaining low attrition while also decreasing dissatisfaction with payment amounts, since motivated VHWs would have the opportunity to earn more through hard work.

To the best of our knowledge, there are no previous studies of PBI for VHWs. Previous research on PBI for other classes of healthcare workers in low income settings has shown mixed effects. A large study in Rwanda demonstrated that facility-based pay-for-performance financing can increase utilization of some maternal and child health services [18]. A PBI system for individual healthcare workers in Cambodia was associated with increased overall healthcare utilization as well as increased utilization of some priority services such as in-facility births [19]. However, other research has suggested that many PBI systems directed at healthcare worker behavior are ineffective, or fall prey to unintended consequences such as diminished intrinsic motivation, adverse selection (e.g., avoiding the sickest patients) or gaming (e.g., exaggerating patients’ severity of illness in order to make improvements look more impressive) [20-22]. The variability in the results of PBI is unsurprising – PBI are a tool that can be implemented in many different ways across a wide range of cultural and institutional contexts. As noted above, this process evaluation assesses issues such as feasibility, acceptability, contextual influences, and other such issues related to implementation. Herein, we do not intend to assess the impact of PBI on healthcare utilization or health outcomes.

Kisoro district is a mountainous area in the southwest of Uganda, nearly 500 km by road from Kampala, the country’s capital. Average educational attainment remains low – only 17.7% of individuals over 15 years of age have completed primary school, and only 4% of individuals over 20 have completed secondary school; 89% of its inhabitants make a living as subsistence farmers [23]. Doctors for Global Health (DGH), Kisoro District Hospital (KDH), and Albert Einstein College of Medicine collaborate to implement community health programs in Kisoro, including a VHW program. In May 2010, they instituted a PBI system, in which VHWs are paid based on completion of a set of priority health activities. Prior to the PBI system, two previous payment systems had been used: first, a monthly salary system, and then a per-visit system, in which VHWs were paid for each household they visited during the month, up to a limit of 80. Under the per-visit system, supervisors did not verify that recorded visits had in fact occurred. In the PBI system, VHWs are paid based on 20 indicators, divided among routine follow-up visits and health education activities, health screening and case identifications, improvements in sanitation coverage, and uptake of priority health services (antenatal care, family planning, cervical cancer screening, and childhood immunization) by patients in their care. A large proportion of VHWs’ salary is based on the PBI system – they do not receive any “base pay” except for a small stipend when they attend training (which can be used to pay for a motorcycle taxi, or can be pocketed by pocketed by VHWs if they travel on foot or bicycle).

Terms such as ‘performance-based incentives’, ‘results-based financing’, and ‘pay-for-performance’ can have a wide range of meanings. Therefore, at this stage it is important to define how we are using the term ‘performance-based incentives’. As the range of payment items suggests, this system contains both activity-based incentives and performance-based incentives. Activity-based incentives are payments for completing a given action, e.g., delivering medication to a patient, or delivering a community education presentation, which only require the VHW to show up. Performance-based incentives are payments that reflect the skill and effectiveness of the VHW, e.g., the VHW’s ability to educate and assist community members in building latrines or attending antenatal care. While this PBI system does not employ measures of health outcomes, we suggest that measures such as sanitation coverage, identification of patients with chronic disease or malnutrition, and the use of immunizations, family planning, antenatal care, and cervical cancer screening services, nonetheless represent key measures of health system performance.

Table 1 summarizes the PBI system used in Kisoro, providing more detail on the individual incentive items. Payment amounts for different items were selected based on three criteria: 1) public health or clinical importance; 2) estimated incidence; and 3) expected difficulty for VHWs. VHWs use a paper-based record system to document their work each month. Eight DGH and KDH staff members work part-time as program supervisors. Supervisors visit each VHW twice a month, providing continued training and clinical support, and making home visits to verify incentive items recorded by VHWs.

**Table 1:** Summary of performance-based incentive system

**Incentive item**	**Amount**	
	**Ugandan shillings**	**Dollars**^**1**^
**Routine visits**		
Visit to patient with acute illness or recent discharge from hospital	500	$0.25
Medication delivery to chronic disease patient	500	$0.25
Annual family information form completed	400	$0.20
Monthly follow-up visit	300	$0.15
**Identification of new patients**		
Chronic disease patient identified	1,000	$0.50
Pregnant woman identified and counseled	1,000	$0.50
Neonate identified and visited	500	$0.25
Malnourished child identified and referred	1,000	$0.50
Disabled child identified and referred	1,000	$0.50
Death identified and family counseled	500	$0.25
‘Difficult home’ identified^2^	500	$0.25
Sanitation risk identified (i.e., home with very poor sanitation)	500	$0.25
**Sanitation facilities**		
New latrine or kitchen constructed in village	2,000	$1.00
New bath shelter, compost pit, or drying rack constructed	1,000	$0.50
**Visits completed by patients (referral and accompaniment)**		
Family planning visit	1,000	$0.50
Cervical cancer screening visit	1,000	$0.50
Antenatal care visit	500	$0.25
Child immunization visit	500	$0.25
**Health education**		
Community talk ≤15 attendees	1,000	$0.50
Community talk >15 attendees	2,000	$1.00
Talk with staff member observing	3,000	$1.50
Attendance at ‘nutrition day’ (malnutrition management outreach)	1,500	$0.75

^1^Based on an exchange rate of 2,000 shillings to $1, the approximate exchange rate when the scheme was designed in early 2010.

^2^‘Difficult home’ refers to domestic violence, child abuse, immunization refusal, or other family problem. It mainly serves as a euphemism for domestic violence or child abuse.

## Methods

### Surveys of VHWs and program supervisors

Anonymous, written surveys were conducted to assess perceptions of the PBI system among VHWs and program supervisors. Both surveys contained a mix of closed and open-response questions. Surveys were distributed to 34 VHWs who had worked in the program since its inception (and thus were familiar with the PBI system as well as previous payment systems), as well as to 8 program supervisors (two of whom had joined the program in the past year, and thus were only familiar with the PBI system). Open-response answers on VHW surveys completed in Rufumbira were translated into English by a DGH staff member and then coded using thematic analysis.

### Interviews of VHWs and program supervisors

Semi-structured interviews were conducted to provide a deeper understanding of issues explored in the surveys. A total of 14 interviews were conducted, 6 with VHWs and 8 with program supervisors. The 6 VHWs were purposively selected to balance age, gender,^a^ and earnings under the PBI system. All 8 program supervisors were interviewed; each interview lasted approximately half an hour. One VHW interview was conducted directly in English; the remaining 5 were conducted in Rufumbira, using a translator fluent in English and Rufumbira. All supervisor interviews were conducted in English. All interviewees provided written consent prior to their interview.

All interviews were audio-recorded and transcribed. For the VHW interviews conducted in Rufumbira, a different translator was used to assist in transcription to verify the accuracy of translation. Interview transcripts were read and re-read multiple times to gain familiarity with the data and identify emerging themes. Thematic content analysis was then used to analyze and summarize the results. Examples of emerging themes were collected and organized. These examples were then re-examined to add additional themes and re-categorize initial themes. Frequencies of themes were also tabulated to provide a way of checking the validity of the emerging themes and guard against ‘cherry-picking’ [24].

### Program costs and attrition

Information obtained from program budget records was used to calculate the costs of using PBI. Costs were estimated for a one-year period from May 2010 to April 2011, which was the first year of operation of the PBI system. Information on VHW attrition from the program was also obtained from program records.

### Quantitative analysis of VHW earnings

Data on VHWs’ annual earnings, productivity, and demographic information were obtained from program records. The distance from each village to the hospital by road was measured using district maps and Google Earth (Google Corp., Mountain View, California, USA). This analysis focused on the effects of VHW and village characteristics on earnings under the PBI system. Analysis was conducted using Stata 11 (Stata Corp., College Station, Texas, USA). Graphs, *t*-tests, and simple linear regression models were used to conduct initial assessments of important associations. Multiple linear regression models were used to adjust for potential confounding variables.

### Project approval

The research presented here was conducted as a component of on-going monitoring and evaluation of a new program. Ethical approval for the publication of this research was obtained from the Montefiore Medical Center Institutional Review Board and the London School of Hygiene and Tropical Medicine Ethics Committee.

## Results

### Surveys of VHWs and program supervisors

Thirty out of 34 VHWs completed the survey, yielding a response rate of 88%; 7 out of 8 (88%) supervisor surveys were returned. To facilitate comparison, the results of both surveys are presented together. Due to the small number of supervisors, fractions rather than percentages have been provided.

When VHWs were asked an open-response question about what they liked about the PBI system, the most common theme was fairness – 13 (43%) respondents included comments about the PBI system distributing payments fairly, based on effort expended. When asked what they disliked about the PBI system, 9 (30%) respondents wrote “nothing”, while an additional 9 (30%) offered complaints about specific incentive items. When asked what they liked about the PBI system, the main benefits mentioned by supervisors were its fairness (5/7), that VHWs work harder (6/7), and that it results in greater health improvements in the villages (5/7). When asked what they disliked about the PBI system, concerns mentioned by supervisors included that VHWs are paid too little (3/7), that it requires too much work by supervisors (2/7), and that it is unfair to VHWs who cover fewer households (2/7).

VHWs and supervisors were also asked to compare the PBI system to the prior incentive system in which they were paid for each household visited per month. When asked to indicate which system is fairer, 22 (73%) VHWs stated that the PBI system is fairer and 7 (23%) stated that the prior system was fairer, with 1 VHW not responding. Of the 6 supervisors who had worked under both systems, all 6 stated that the PBI system was fairer. When VHWs were asked which payment system they felt improves health more, 25 (83%) stated that the PBI system improves health more, 2 (6%) stated that the prior system improved health more, and 3 (10%) did not answer. When asked why they believe that the chosen system improves health more, of those choosing the PBI system 5 (20%) mentioned that VHWs do more work under the PBI system, 9 (36%) mentioned that supervisors are more involved under the PBI system, and 5 (20%) specifically mentioned sanitation improvements in their villages. Of the 6 supervisors who worked under both systems, all 6 said the PBI system improves health more. When asked why, they stated that VHWs work harder under the PBI system (4/6), and that supervisors provide more medical treatment in the villages under the PBI system (3/6). Finally, VHWs were asked under which system they earned more money; 17 VHWs (56%) estimated that they earn more money under the PBI system, 6 (20%) estimated they earned more under the prior system, and 7 (23%) did not select an answer.

### Interviews of VHWs and program supervisors

Six purposively selected VHWs were interviewed, all of whom have served as VHWs since the program’s inception. Emerging themes were grouped in three main areas: 1) perceptions of fairness of the PBI system, payment amounts, and volunteerism; 2) non-financial incentives; and 3) relationships with their communities. For both the VHW and supervisor interviews, individuals are given numbers to demonstrate when the same individual is quoted repeatedly.

VHWs viewed the PBI system as fair due to the perceived lack of forgery under this system, compared with the acknowledged forgery of household visits under the prior incentive system (i.e., VHWs would note in their records that they had visited a household without actually making the visit). Four of the 6 VHWs interviewed mentioned that they liked the PBI system because VHWs are “*paid for the work done*”, or another similar phrase. Two VHWs explicitly contrasted this characteristic to the forgery that occurred under the prior system. VHW 5 explains:

“*Unlike the old system, where most VHWs* [village health workers] *would be paid for things they had not done – not all of us, but some of us, would forge…This system is genuine – there is nothing like mistrust. It is genuine – you are paid for what you did*”.

Thus, VHWs seemed to feel that it was important for a payment system to reward hard work (while paying less to those who did not work as hard).

However, VHWs did not always feel that the PBI system sufficiently rewarded them for effort expended. Three out of 6 VHWs expressed frustration at putting in effort looking for case identifications (i.e., conducting screening for malnutrition, chronic disease, etc.) without finding anything for which they would be paid, or about conducting unreimbursed follow-up visits. VHW 1 explains:

“*Sometimes you move, like doing sensitization* [education]*, and other things, you move, you don’t find some things to record. But of course you have done* [something]*, because you have spent time in the village*”.

All the VHWs interviewed also expressed a desire to be paid more. Dissatisfaction with payment amounts was driven in part by the inflation that Uganda experienced in early 2011. Four out of 6 VHWs mentioned the issue of inflation as an important concern.

Perceptions of fair recompense are of course tied to the VHWs’ willingness to volunteer their time. All VHWs interviewed considered themselves voluntary workers rather than employees, a distinction they felt was important for their relationship with their communities. They also emphasized that they did not view their work as VHWs as a career path or as a primary income source, but rather as an avocation undertaken for the benefit of their communities. However, they did not feel capable of offering a great deal of volunteer time due to their other work and obligations to their families. Three VHWs described the opportunity cost of serving as VHWs as a challenge. VHW 4 explains:

“*You also realize that when you devote 3 days to village health work, it is some kind of loss because you are not doing other things*”.

VHW 1 offers a more nuanced description, expressing the same concept of an opportunity cost:

“*We take much time in the village, in the community, trying to move door to door, looking for the patients, trying to sensitize some of the people, which does not allow us to do our personal work. And in return, the money given to us does not accommodate the work we would be doing at home…So that’s the challenge, isn’t it*”.

VHW 5 also presented the issue as a direct opportunity cost, suggesting that VHWs should be paid a stipend equivalent to what a day laborer would receive for working in the fields, so that they could hire someone to till their crops while they conducted their work as VHWs. Together, these responses suggest that VHWs perceive themselves neither as fully volunteers nor as salaried employees. None of the VHW expressed a desire to progress to full-time healthcare work, or be paid a salary equivalent to a healthcare worker. However, they felt strongly that they should be compensated for the time that they would otherwise spend farming.

VHWs also discussed a number of intrinsic motivations for their work. Five out of the 6 VHWs mentioned that they enjoyed the knowledge and skills they gained from training sessions, and the status this training gave them in their villages. Two VHWs also commented that this knowledge benefited them and their families by allowing them to maintain their own health. VHW 6 commented:

“*Ever since I joined I acquired a lot that helped improve my household…in case I get a disease, I have skills so I know how to handle it before I get very sick*”.

All 6 VHWs mentioned the benefit to their community as an important motivating factor for their participation. VHW 3 noted:

“*What makes me happy as a VHW is that I help community members who elected me, and who trusted me, and I help them in health issues*”.

VHWs discussed a range of benefits to the community, focused mainly on clinical services and sanitation improvements. VHWs also emphasized that close supervision under the PBI system was beneficial – 5 out of 6 VHWs mentioned that the education and clinical work of supervisors increased their status in the village or increased the community’s trust in them. One VHW explained:

“*Even if the supervisor teaches the same things as the VHW, then the communities will say, yeah, the VHW was teaching things that she knows*”.

VHWs offered varying perspectives on whether being paid affected their relationship with their villages. All VHWs felt that payment was a sensitive issue, and took pains to emphasize to their communities that they were volunteers rather than salaried employees. Two VHWs felt that being paid did not affect their relationship with the community, because they were still perceived as volunteers. VHW 3 explained:

“*The community members are not even aware that we are paid. Community members know that we are voluntary workers, and we are not working for the government, so whatever stipend we get is between us and the supervisors*”.

However, the other 4 VHWs seemed to feel that being paid did create tension in their relationship with their villages. VHW 6 explained:

“*Most* [people] *are happy because of the work we are doing for them. The services we offer make them feel happy. But maybe some people say that these* [VHWs] *are working for their pay, so if you give them time you are giving them more money…You know that jealousy is always there in the community*”.

Though the VHWs’ services are provided to the community for free, the knowledge that VHWs have found part-time paid employment appears to engender resentment nonetheless.

All 8 program supervisors were interviewed; 4 of the supervisors are healthcare workers at KDH and the remaining 4 are DGH staff members without clinical training. The interview results from both groups have been presented together in order to preserve anonymity.

Overall, supervisors expressed a higher level of satisfaction with the PBI system than VHWs. Supervisors perceived the PBI system as fairer than the prior incentive system due to the lack of forgery. Six of the 8 supervisors mentioned the concept of the PBI system being ‘results-oriented’, VHWs being “*paid for the work done*”, or another similar phrase. Supervisor 7 explained:

“*I think the new* [PBI] *system is very genuine. Because if you work hard, then you earn more. If you work less, then you get less. And it’s the most fair* [system]”.

Even more than VHWs, the supervisors were insistent about the problem of forgery under the prior incentive system. Five of the 6 supervisors who had worked under the prior system asserted that there had been forgery of VHW records. All 8 supervisors were similarly insistent that the PBI system prevented this kind of forgery because the verification process provided little opportunity for it. Supervisor 3 explained:

“*This is actually what is observed on the ground. We can’t stipend something that we’ve not seen. We have to first observe what is done. If it is a pregnant woman, we have to first see that one. If it is a toilet, we have to see that one and see that it is a verified one and then we give a stipend for that. So I believe there is no faking*”.

Several supervisors also mentioned that they felt the PBI system gave them a more substantive role during supervision, as well as increasing the community’s trust in the VHW.

Despite their satisfaction with the lack of forgery, 5 of the 8 supervisors expressed some discomfort with what they perceived as the competitive nature of the PBI system or the possibility that VHWs would expend effort without a definite reward. Three supervisors mentioned the wide distribution of payment amounts under the PBI system, saying that it discouraged the VHWs who earned less. Two supervisors also mentioned that it is discouraging or unfair for VHWs to put in substantial effort searching for new case identifications (i.e., conducting screening) without necessarily being rewarded. Supervisor 2 explained:

“*You know, to identify an item that will be rewarded. It takes some hours. You can imagine someone, moving from home to home looking for a pregnant mother. Suppose he fails to get a pregnant mother, goes home, and those hours are not rewarded…So it’s* [the PBI system] *good, but somehow it’s tricky*”.

In addition to this concern about the uncertain rewards provided by the PBI system, 5 of the 8 supervisors also mentioned that they felt VHWs should be paid more.

### Program costs

The total annual cost of the VHW program using the PBI system was $18,000 (from May 2010 to April 2011). The main areas of expenditure were the VHW incentives (27.5%), training costs (30.5%), and supervision (27.9%), with administrative costs adding an additional 12.2%. The paper forms used for record-keeping constituted an additional 1.8% of the total, though they were donated and did not represent an actual outlay by DGH. The total cost per VHW was $516, with each VHW serving an average of 115 households. Based on the average household size in Kisoro district [23], this cost equals $1.12 per individual served by the program per year. Table 2 provides a more detailed breakdown of the program costs.

**Table 2:** **Annual program expenditures**^**1**^

**Category**	**Item**	**Annual cost ($)**^**2**^
VHW stipends	VHW stipends	$4,953 (27.5%)
Training	Materials (incl. VHW lunch, snack, and transportation refunds)	$3,795
	Facilitator pay	$891
	Standardized patient program	$805
	*Total*	$5,490 (30.5%)
Supervision	Supervisor pay	$3,251
	Transportation	$1,779
	*Total*	$5,030 (27.9%)
Administration	Staff pay	$1,856
	Cell phone airtime	$340
	*Total*	$2,195 (12.2%)
Donated costs	Program forms	$331 (1.8%)
Total costs	Annual total cost	$18,000 (100%)
	Annual total cost – per VHW	$516
	Annual total cost – per household served	$4.61
	Annual total cost – per individual served^3^	$1.12

^1^For VHW stipends, actual expenditures were used. For all other categories, budgeted expenditures were used. This will tend to overestimate training costs, since it assumes 100% attendance at training.

^2^An exchange rate of 2,120 Ugandan shillings per dollar has been used, the exchange rate as of May 1, 2010 (historical exchange rate obtained from: XE Current and Historical Rate Tables [http://www.xe.com/currencycharts/?from=USD&to=UGX&view=2Y], accessed July 5, 2011).

^3^Population of villages served estimated using the average household size for Muramba subcounty (taken from: Uganda Bureau of Statistics, 2002 Uganda Population and Housing Census: Kisoro District Report. Entebbe: Uganda Bureau of Statistics, November 2005).

### VHW attrition

Overall, attrition has been low. The program began with 40 VHWs; during the first three years (prior to the PBI system), 4 VHWs quit or were dropped from the program due to poor performance. During the year in which the PBI system was implemented, one VHW died unexpectedly and another was dropped from the program due to poor performance and unprofessional behavior.

### Quantitative analysis of VHW earnings

The dataset analyzed here included 34 VHWs; 15 (44%) were male and 19 (56%) were female. VHWs had completed an average of 8.9 years of schooling; 12 VHWs had attended some or all of primary school (but no secondary school), 18 had attended some secondary school, and 4 had completed secondary school. On average, males had completed slightly more formal education than females (a mean of 9.3 vs. 8.5 years).

VHWs were responsible for an average of 115 households, which translates to an estimated average of 471 individuals, based on the average household size in Kisoro district [23]. The average distance to the district hospital (KDH) by road was 9 km. Table 3 displays the mean, standard deviation and range for each of the continuous variables mentioned above.

**Table 3:** Characteristics of VHWs and their villages

**Measure**	**Mean**	**Std. dev.**	**Min**	**Max**
Years of education	8.9	3.1	2	15
Households served	115	52.7	63	280
Est. population served	471	216	258	1,148
Distance from hospital (km)	9.0	2.6	4.6	12.7
Annual pay ($)	144	65	40	306

**Figure 1:**
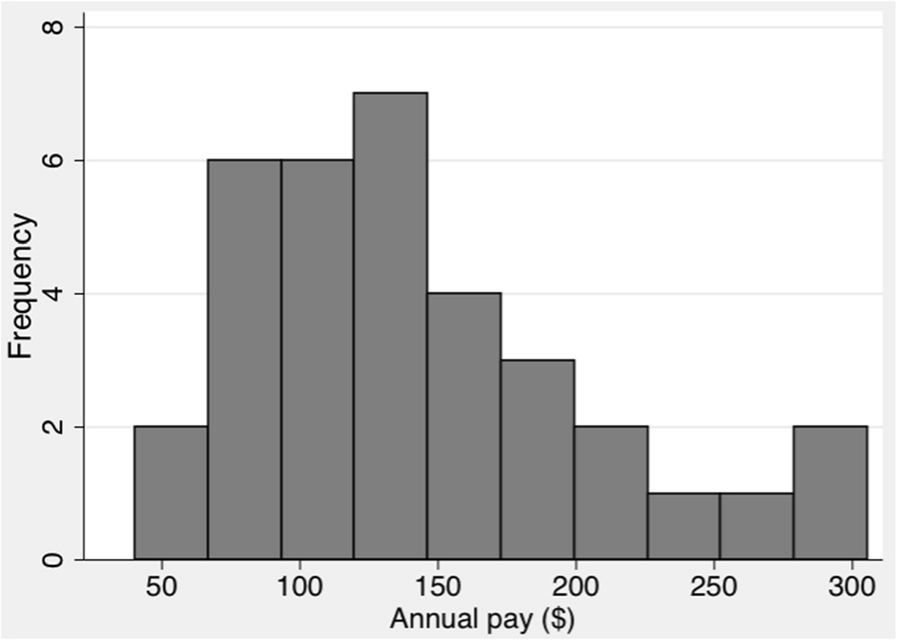
Histogram of annual pay.

The mean annual pay was $144 ($12 per month). Figure 1 displays a histogram of annual pay, demonstrating a slightly right-skewed distribution and a wide range of payment amounts. For the first year of operation of the PBI system, the average annual payment was essentially identical to earnings under the prior incentive system. However, the PBI system resulted in a greater range of payments – the highest-earning VHWs earned 3 to 5 times as much as the lowest-earning VHWs.

The impact of VHW and village characteristics on earnings under the PBI system has implications both for the fairness of the system and the selection of VHWs. We examined the relationships between annual pay and number of households served, gender, education level, and distance from the hospital.

#### Number of households

The number of households for which a VHW is responsible is a strong predictor of annual pay. Simple linear regression confirms the strength of the association (*t* = 4.89, *P* <0.001); the strength of the association persists when adjusting for potential confounding. The details of the regression model results for all variables considered are given in Table 4.

**Table 4:** **Regression results for annual pay**^**1**^

**Explanatory variable**	**Coefficient**	**Std. error**	***t*-value**	***P*** **> | *t *** **|**
**Number of households**				
Number of households	0.800	0.164	4.89	<0.0001
Constant	52.6	20.6	2.55	0.016
r-squared	0.427			
Adjusted r-squared	0.409			
**Education**				
Years of education	–0.127	4	–0.03	0.973
Constant	146	34	4.23	<0.001
r-squared	0			
Adjusted r-squared	–0.0312			
**Distance from hospital**				
Distance from hospital (km)	4.91	4.25	1.15	0.257
Constant	100	39.9	2.51	0.017
r-squared	0.0400			
Adjusted r-squared	0.0100			
**Full adjusted model**				
Number of households	0.849	0.180	4.66	<0.0001
Female gender	36.3	17.2	2.11	0.044
Education	0.462	3.08	0.15	0.882
Distance from hospital (km)	–1.12	4.05	–0.28	0.783
Constant	34.0	54.1	0.63	0.535
r-squared	0.512			
Adjusted r-squared	0.445			

^1^The original data for annual pay was in units of Ugandan shillings. An exchange rate of 2,120 shillings to $1 was used for this conversion, the exchange rate as of May 1, 2010 (historical exchange rate obtained from: XE Current and Historical Rate Tables, [http://www.xe.com/currencycharts/?from=USD&to=UGX&view=2Y], accessed July 5, 2011).

#### Gender

The mean annual pay for women was $159, compared with $126 for men. A *t*-test suggested that this difference could be due to chance (*t* = –1.52, *P* = 0.1381; full results not shown in Table 4), though adjusting for education, distance from the hospital, and number of households using a multivariable regression model strengthened the evidence for an actual correlation between gender and annual pay (*t* = 2.11, *P* <0.044).

#### Education level

Simple linear regression showed no association between level of education and earnings under the PBI system (*t* = –0.03, *P* = 0.973). Adjusting for possible confounders produced little change in the result.

#### Distance from the hospital

VHWs in villages further from the hospital tended to earn more, though the association was not statistically significant (*t* = 1.15, *P* = 0.257). Furthermore, the slight association was likely due to confounding with number of households, since VHWs further from the hospital covered, on average, more households. The adjusted multiple regression model showed no indication of an association (*t* = –0.28, *P* = 0.783).

Finally, the PBI system facilitates easy measurement of VHWs’ productivity. Among other activities, in the annual data examined here, VHWs identified and referred 210 malnourished children, identified and counseled 791 pregnant women, visited and examined 679 neonates, referred or accompanied 188 women for new family planning uptake, and completed 5,672 home visits to patients with an identified need.

## Discussion

In this assessment of PBI for VHWs, we found overall satisfaction with the PBI system was high, though both VHWs and supervisors also reported specific concerns about the PBI system – most notably dissatisfaction with payment amounts. Attrition from the program was fairly low both before and after implementation of the PBI system. Our quantitative analysis of VHW earnings suggests that the PBI system operates fairly with respect to education level, gender, and proximity to the district hospital.

Previous research suggests that dissatisfaction with payment amounts represents one drawback of monetary incentives [5,16,17]. In this evaluation, both supervisors and VHWs felt that the PBI system distributed payments according to the amount of work accomplished. However, both VHWs and supervisors agreed that VHWs should be paid more. VHWs expressed dissatisfaction with both the overall level of payment as well as with the uncertainty of reward for case-finding (i.e., that screening did not always lead to case-finding). These concerns may reflect decreased intrinsic motivation in the setting of PBI. However, VHWs also expressed dissatisfaction with their pay under the prior incentive system (in which they were paid per household visited). Most VHWs in fact believed they were earning more under the PBI system, suggesting that this dissatisfaction with payment amounts is not specifically linked to a PBI system. We were unable to assess whether VHWs’ dissatisfaction with payment amounts adversely impacted the quality of their work, or merely represented an expression of the near-universal human desire to be paid more. However, the low level of attrition perhaps suggests that the VHWs’ dissatisfaction with payment amounts has not significantly impacted the success of the program.

In response to these results, DGH and KDH have added additional incentives for routine data collection and training performance, increasing the average payment level to $20 per month ($240 per year). Future program monitoring and evaluation will reveal whether this increase resolves VHWs’ concerns about payment amounts or merely establishes a new set point from which to bargain.

VHWs expressed mixed views about volunteerism in the program. VHWs felt they should be compensated well enough to ensure that the time they spent on their work as VHWs did not have a negative effect on their families’ wellbeing. However, they framed their request as a desire to paid at the same level as an unskilled farm laborer, suggesting some level of volunteerism, since they were not seeking payment at the level of the ‘market value’ for their skills. In some VHW programs, the desire for future, better-paid employment is a key motivating factor [13,16]; this did not appear to be the case in the program evaluated here. All VHWs interviewed mentioned intrinsic motivations, such as knowledge gained and the satisfaction of seeing health improvements in their village, as reasons for their participation. However, the fact that VHWs are paid emerged as a potential area of friction between VHWs and their communities due to jealousy among some community members that the VHWs had secured part-time paid employment.

The PBI system requires close supervision of VHWs, which has the benefit of bringing clinicians into the communities. Close supervision also enables the continuous collection of well-validated data via the PBI system, creating the potential for rigorous program monitoring. The benefits of the PBI system described by supervisors and VHWs may have resulted in part from the closer supervision built into the PBI system. In one sense, this represents an obstacle to assessing the PBI system, since it is difficult to disentangle the effect of the PBI system from the effect of closer supervision. In another sense, as noted in the interviews with supervisors, closer supervision is facilitated by the PBI system, in that the PBI system provides greater structure for the supervisory role. Previous research suggests that close supervision is critical to VHW program functioning [5]. Regardless of the incentive model selected, we suggest that VHW programs are unlikely to be successful without providing frequent supervision. In this light, it is encouraging that the actual incentive payments comprised less than 30% of total program costs.

The quantitative analysis of VHW earnings reveals several notable findings. VHWs with greater education earned no more than those with less education, suggesting that VHWs with limited formal education can master a PBI system. Contrary to our expectations based on gender inequality [25], female VHWs out-earned male VHWs under the PBI system. The success of female VHWs under the PBI system may stem from greater effort expended, or from the greater trust that other women place in them (since many of the incentives pertain to maternal and child health). We hypothesized two possible effects of proximity to the hospital: that VHWs in more distant villages would find it more difficult to refer patients for services mainly available at the hospital (such as family planning and cervical cancer screening), or that people in more remote villages would have less access to other health services and would rely more on their VHW. However, we did not find evidence of an association between proximity and VHW earnings under the PBI system.

These results suggest that the PBI system operates in a fair way, at least with respect to the demographic characteristics of VHWs and their villages. Payment amounts were higher for VHWs with more households. While this could suggest that it is easier for these VHWs to succeed under the PBI system, it seems more likely that VHWs serving more households must expend more time and effort in order to care for a larger population. Furthermore, if the PBI system acts as an accurate measure of performance, then VHWs in larger villages are simply being paid for greater performance (e.g., helping a greater number of people gain access to services such as improved sanitation, family planning, malnutrition treatment, etc.).

This work has a number of limitations. The most significant limitation is that we evaluated a relatively small VHW program over a relatively brief period of time, within a specific programmatic and cultural context. Thus, this process evaluation serves as an initial overview of a new system rather than any sort of definitive assessment. In addition, while this evaluation allowed us to assess issues such as the feasibility and acceptability of PBI for VHWs, it does not provide information on outcomes. Due to the data available, we are unable to assess the impact of the PBI system on utilization of priority health services in Kisoro relative to other possible payment systems. As the productivity data presented in the results demonstrate, the PBI system generates metrics (such as malnutrition case-finding and treatment) that could be used as outcome metrics. However, we did not have data for any of these metrics prior to the implementation of the PBI system, preventing a before-after comparison. A larger evaluation with the resources to prospectively track case-finding and utilization of care would be needed for an impact evaluation.

The specific components of this research also have their own limitations. Some VHWs have only basic literacy, and may have had difficulty in fully comprehending the questions on the survey. One potential limitation of the interviews is that they reflect the views of only 6 VHWs. Although there was substantial thematic overlap across the 6 interviews, it is possible they do not represent the full range of views. The complex issue of reflexivity also poses potential problems. The first author previously spent a year as a DGH volunteer in Kisoro, Uganda, working on a variety of community health programs including the VHW program. Thus, it is possible that VHWs and supervisors were reluctant to offer criticism to someone they viewed as a colleague. Alternatively, given the prevailing culture of politeness and group solidarity in Kisoro, it is also possible that VHWs and supervisors would be more willing to offer criticism to someone they knew than to an outsider. It is also possible that the VHWs viewed the surveys and interviews as an opportunity to lobby for greater pay, and exaggerated that concern. The small number of VHWs in this program also limited the power of statistical tests used in the quantitative analysis. This process evaluation also did not include any evaluation of community perspectives on the program and the PBI system.

Further research is also needed to assess the long-term impact of PBI incentives, and particularly whether they diminish intrinsic motivation over time and, if so, whether diminished intrinsic motivation harms program functioning. Considerable evidence in the psychological and economic literature suggests that extrinsic rewards undermine intrinsic motivation [26,27]. However, it is unclear whether this research applies to long-term employment, or whether different payment schemes for VHWs (e.g., salary vs. PBI) would have differing effects on intrinsic motivation. An additional concern presented in the literature suggests that PBI may lead to distortions, in which workers focus on incentivized activities and neglect non-incentivized activities [28,29]. While this evaluation did not specifically address this issue, we suggest that distortions are less likely in PBI for VHWs than for other healthcare workers, since the more limited scope of practice of VHWs makes it easier to create a comprehensive PBI system that incentivizes many if not all of the desired activities of VHWs. In addition, if incentive amounts for different accomplishments are accurately calibrated with the health benefits, then VHWs will have an incentive to allocate their effort appropriately. Incentive amounts can also be adjusted at different times of the year based on seasonal variation in health needs, e.g., increasing incentives for malaria prevention and diagnosis in the rainy seasons. Incentive amounts can easily be adjusted upward over time to keep pace with inflation or to reward increasingly long-serving and experienced VHWs, just as salaries might increase over time. However, assessment of these issues will require long-term follow-up.

## Conclusions

This process evaluation demonstrates that a PBI system can be motivating, acceptable, and feasible for VHWs and supervisors, at least in short-term follow-up and within a particular cultural and institutional context. However, the longer-term effects of the PBI system remain uncertain. VHWs with limited formal education were able to master the PBI system and perform as well as colleagues with more formal education. A PBI system requires close supervision of VHWs and detailed record keeping. Apart from the impact on VHWs and supervisors, a main strength of PBI is that the operation of the incentive system automatically generates useful data. PBI thus have the potential to improve monitoring and evaluation by providing objective, verified metrics of program performance. Further evaluation is needed to assess the scalability and transferability of PBI for VHWs, as well as the impact on outcome measures.

## Endnote

^a^We use the word “gender” rather than “sex” throughout this paper, since the hypothesized differences in earnings relate to the social structure of Ugandan society rather than any biological characteristics.

## Abbreviations

DGH: Doctors for global health; KDH: Kisoro district hospital; PBI: Performance-based incentives; VHW: Village health worker.

## Competing interests

The authors declare that they have no competing interests.

## Authors’ contributions

JSM and GAP conceived the study. JSM collected data, performed the data analysis, and drafted the manuscript. SM and MB assisted in data collection. All authors participated in refining the study design, assisted in revision of the manuscript, and approved the final manuscript.
